# Patient perspectives on endoscopic sinus surgery for chronic rhinosinusitis

**DOI:** 10.1186/s40463-021-00515-z

**Published:** 2021-06-15

**Authors:** Nadim Saydy, Sami Pierre Moubayed, Martin Desrosiers

**Affiliations:** 1 Division of Otolaryngology – Head & Neck Surgery, Centre Hospitalier de l’Université de Montréal, University of Montreal, 1051 Sanguinet Street, Montreal, QC H2X 3E4 Canada; 2 Division of Otolaryngology – Head & Neck Surgery, Sacré-Coeur Hospital, University of Montreal, Montreal, Quebec Canada

**Keywords:** Chronic rhinosinusitis, Endoscopic sinus surgery, Patient-centered care, Qualitative research

## Abstract

**Background:**

Through shared decision-making, physicians and patients can elect endoscopic sinus surgery (ESS) when maximal medical therapy fails in patients with chronic rhinosinusitis (CRS). In this study, we aim to explore the most important themes with regards to patients’ perspectives on ESS. Our objective was to define the patient experience and ensure that we have congruent physician and patient goals for obtaining success.

**Methods:**

Semi-structured face-to-face interviews were conducted with 22 patients at a tertiary-care institution in Montreal. Three themes were established a priori: living with CRS, objectives and expectations and criteria for success. This thematic approach allowed the identification, analysis and reporting of patterns found across the data set. A phenomenological methodological orientation was used. Interviews were audio-recorded and transcribed verbatim for continuous analysis. These were coded by hand by a single coder who read the transcripts multiple times and relistened to the recordings.

**Results:**

Exploration of themes on patients’ perspectives on ESS for CRS yielded multiple anecdotal findings, and some recurring patterns. There is a tendency for patients to focus on one principal symptom that drives their decrease in QoL. Headaches and nasal congestion seemed to impact patients’ QoL the most amongst rhinologic symptoms. Hyposmia was rarely spontaneously by patients but was often a significant source of distress when prompted during interviews. Objectives and expectations seemed to be inversely proportional to number of previous surgeries and severity of symptoms preoperatively. There was a clear association between preoperative expectations and postoperative satisfaction. There was no clear pattern in the improvement magnitude or time improved postoperatively for patients to consider the surgery a success.

**Conclusions:**

Patients’ level of satisfaction postoperatively and with their care in general is multifactorial. We believe the topic of goals and expectations regarding ESS should be discussed preoperatively for every patient with CRS. This includes patients with seemingly minor disease and patients naive to surgery, as can sometimes have exceedingly high expectations. Preoperative counselling must also include an assessment of what symptom is the most cumbersome to that particular patient, as patients tend to focus a lot on one or two symptoms. Postoperatively, we encourage clinicians to be attentive to the change in each patient’s principal complaints within the context of a personalized approach and to refer back to patients’ preoperative goals in their assessment of operative success.

**Graphical abstract:**

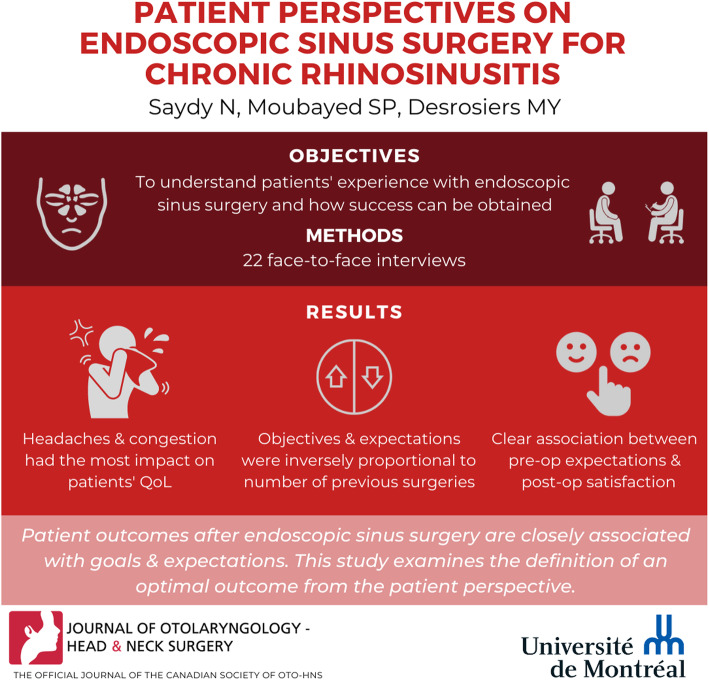

## Introduction

Chronic rhinosinusitis (CRS) is characterized by inflammation of the nose and paranasal sinuses. Its diagnosis is incumbent upon both subjective complaints and objective findings (endoscopy or CT scan). The *EPOS 2012 Position Paper on Rhinosinusitis and Nasal Polyps* defines CRS as two or more of the following symptoms for at least 12 weeks: nasal blockage/congestion/obstruction, nasal discharge, facial pain/pressure, hyposmia/anosmia (with the presence of at least either nasal blockage, congestion, obstruction or nasal discharge) [1]. These rhinologic symptoms are often an important burden on patients’ quality of life (QoL). Beyond these symptoms, multiple studies link CRS with sleep disturbances, fatigue, depression, anxiety and an overall decrease in quality of life [2–5]. While rhinologic symptoms are sufficient for the clinical diagnosis of CRS, non-rhinologic complaints remain an important part of the clinical portrait. A recent study has shown that patients with depression have an increased sino-nasal disease burden and pain compared to non-depressed patients. In fact, surgical outcomes appear less favorable in the former group [6]. A thorough understanding of patients’ perspectives and experiences with CRS and endoscopic sinus surgery (ESS) is likely to favor patient-physician collaboration and improve shared decision-making.

The *Quality Improvement Committee of the American Rhinologic Society* identified CRS as a priority in the development of quality measures for rhinologic diseases [7]. One significant obstacle in all aspects of the management of CRS remains the ability to have universal, patient-centered definitions of commonly used terms. For example, there is no clear definition of what an optimal surgical outcome after ESS is, partly because even experienced clinicians do not fully understand each patient’ perspective and overall experience. There is also no unique agreed-upon definition for an “acute exacerbation” of CRS, as Wu et al. showed in their systematic review published last year [8]. It is exceedingly complex to perfectly pinpoint why each patient wishes to undergo surgery for CRS, but perhaps there are common themes. More and more, effort in research in put towards defining important concepts in Rhinology and developing clinical recommendations based on evidence. Last year, the *Rhinology Subspecialty group of the Canadian Society of Otolaryngology – Head & Neck Surgery* published recommendations for the diagnosis and treatment of acute rhinosinusitis and nasal fracture through the *Choosing Wisely Canada campaign* [9]. This initiative is primarily aimed at reducing unnecessary tests and treatments, by giving clinicians the tools to assist patients in shared decision-making.

With improving surgical techniques and novel medical therapy, the outcomes of CRS patients have steadily been improving. That said, there remains a small subset of patients who do not experience a significant improvement in their CRS symptoms postoperatively, or who only get a short-lived improvement in their QoL [10]. An in-depth understanding of patients’ experiences and views is paramount to the patient-physician partnership. Being familiar with patients’ journey would help clinicians improve their therapeutic relationships with patients, identify causes for dissatisfaction and predict post-operative disappointment. This study aims to broadly further our understanding of patients’ experiences and views on CRS and ESS.

## Methods

### Study design

A qualitative research methods was chosen for this study. Gallo et al. state that “this type of research has the potential to enhance the understanding of surgeons’ and patients’ preferences, attitudes and beliefs, as well as assess how these may change with time” [11]. Study methodology and findings are reported in accordance with the Consolidated Criteria for Reporting Qualitative Research (COREQ) [12]. A phenomenological methodological orientation was used; namely, the focus of the study was on individual subjective experiences. Three themes were identified a priori and incorporated into the interview template: living with CRS, objectives and expectations and criteria for success. Informed written consent was obtained from all participants before entry into the study. This study was approved by the University of Montreal Healthcare Center Institutional Review Board and was conducted following study protocol and the principles of the Declaration of Helsinki.

### Setting

Twenty-two participants were recruited from a single surgeon’s tertiary care practice (Rhinology & Skull Base Surgery) between August 2018 and January 2019 at the University of Montreal Healthcare Center (CHUM) clinic of Otolaryngology – Head & Neck Surgery. Participants were adult patients (≥ 18 years old) with either CRSwNP (Chronic Rhinosinusitis with Nasal Polyposis) or CRSsNP (Chronic Rhinosinusitis without Nasal Polyposis) who had undergone at least one ESS. Patient charts were accessed before the start of clinic to determine eligibility. Participants were purposively approached face-to-face before their scheduled appointment in the waiting room. Interviews were conducted either before or after their visit, depending on clinical workflow. Before initiating interviews, the interviewer explained to patients he had training as a physician but was not part of the medical team. Short-term and long-term objectives of our study were explained to participants; it was stated that data would be used for scientific research, rather than for internal audit purposes. A total of 27 patients were approached, only 3 declined to participate. In all 3 cases, it was because their schedule did not permit it.

### Interview

Interviews were conducted in a semi-structured, face-to-face manner by NS (male), who at the time of the study held a Medical Degree and was enrolled in a full-time Masters’ Degree in Biomedical Science (Clinical Research) at the University of Montreal. Interviews were scheduled to last 15 min. Based on a review of literature, an interview template composed of 8 open-ended questions was produced [13–16]. An iterative process was conducted throughout interviews to remove low-yield questions or add prompts; the final version is shown in Appendix 1*.* Specific prompts were added to stimulate discussion and provide a structure. Moreover, this allowed participants to provide impressions they had not spontaneously mentioned. Each question was evaluated by the senior author (MD); items were subtracted, and wording was modified to ensure that concerns commonly raised by patients during clinical visits were addressed. Issues identified in early interviews were corrected for subsequent interviews. Whenever patients did not understand the question, the interviewer provided clarification or reformulated the question. Each participant was interviewed once. Patients were recruited until thematic saturation was achieved. All interviews were conducted by the same interviewer. Interviews were conducted either in French or in English, depending on patient preference.

### Analysis

Data saturation was achieved after 22 interviews, which prompted us to stop the interview process. Sample size in qualitative studies is determined when data saturation is achieved [17]. This is determined during data collection, when no new information or themes are discussed during interviews [18]. Thus, there is no formula or criteria to determine sample size, but some authors propose 12 to 26 interviews as a general rule of thumb [19]. Interviews were audio-recorded and transcribed verbatim for continuous analysis. Transcripts were coded by hand and were not returned to patients for comments or corrections. No data extraction software was used. Feedback was not provided to participants. There was one coder (NS), who listened repeatedly to the recordings and reread the transcripts. A thematic approach was used to identify common patterns among the interviews. This allowed the identification, analysis and reporting of patterns found across the data set.

## Results

The average interview time was 13.3 min (range: 8–25). The median age was 53 years old (range: 19–79) and there 14 male participants (64%). The median number previous ESS was 2 (range: 1–7). Seventeen patients had CRSwNP (77%). Many patients had severe forms of CRS, with recurring disease despite surgical intervention and maximal therapy. Patients had many comorbidities including asthma, aspirin intolerance and atopy. One patient had a concurrent diagnosis of common variable immune deficiency. Most patients were not active smokers (77%) and the majority of patients were Caucasian (90%). Demographic data and disease characteristics for interviewed participants are presented in Table 1.

**Table 1:** Participant demographic and disease characteristics (*n* = 22)

**Male gender**; *n (%)*	14 (64%)
**Age**; *median (range)*	53 (19–79)
**CRSwNP**; *n (%)*	17 (77%)
**Number of previous ESS**; *median (range)*	2 (1–7)
1	9 (41%)
2–4	12 (55%)
5+	1 (5%)
**Months since last ESS**; *median (range)*	12 (0.25–180)
**Asthma**; *n (%)*	9 (41%)
**Intolerance to ASA**; *n (%)*	6 (27%)
**Atopy**; *n (%)*	9 (41%)
**Immune deficiency**; *n (%)*	1 (5%)
**Ethnicity**; *n (%)*	
Caucasian	20 (90%)
African American	1 (5%)
Middle Eastern	1 (5%)
**Smoking status**; *n (%)*	
Never	12 (55%)
Former	6 (27%)
Active	4 (18%)

*ASA* Aspirin; *CRSwNP* Chronic rhinosinusitis with nasal polyposis; *ESS* Functional endoscopic sinus surgery

### Living with CRS

Many patients reported nasal congestion as the most bothersome symptom. They also reported secondary sleep disturbances, as well as an impact on activities of daily living (including sports). According to them, these were consequences of the nasal congestion. Patients with diagnosed sleep apnea who were dependent on a CPAP machine reported difficulty using it because of their inability to breathe through their nose. One participant was a competitive weightlifter. The obstructive symptoms became so severe that he could not exercise anymore and had to undergo surgery only to continue training.

*When it was completely blocked before surgery, I could not breathe at all. At night, my mouth got very dry. If I choked on food, I could not breath from the nose.*

(Participant 16, male, CRSwNP)

*Try blocking your nose with a clothespin for 24h, I dare you. Then imagine that for years.*

(Participant 16, male, CRSwNP)

Patients seldom described a single symptom and most often had multiple rhinologic complaints before ESS. They could usually identify a principal symptom mostly responsible for the decrease in QoL. In many cases, that symptom was the persistent and incapacitating headaches. Patients with CRSwNP were also very bothered by the severe nasal congestion.

*With sinusitis, it’s not only the headaches …*

*I always feel like I’m in a box, like my head is in a box.*

(Participant 17, female, CRSwNP)

When asked to describe what it feels like to have CRS, some patients reported symptoms which are not traditionally reported in CRS as being part of their disease, such as hypoacusis, pruritus or tinnitus.

*It’s itchiness on the nose, it’s an uncomfortable squeezing sensation.*

*It’s as if someone is lifting you from the tip of your nose.*

(Participant 7, male, CRSwNP)

Hyposmia and anosmia were rarely mentioned spontaneously as a symptom of CRS. Only with additional prompts did many patients describe anosmia, which often had a tremendous impact on their QoL. One participant was passionate about cooking and drinking wine and described anosmia as a “handicap”. Three patients also mentioned being afraid of eating leftover food because they could not tell if it was still edible without a normal sense of smell and taste. One patient also noticed an association between acute sinusitis episodes and a feeling of cacosmia.

*My sinusitis greatly affected my quality of life. Even working full-time was difficult. I could not eat; I could not smell. I was afraid of tasting food from fear of getting sick. I could not smell fire smoke. I could not smell anything.*

(Participant 13, female, CRSwNP)

Anterior and posterior nasal drip were also described by patients as a frustrating symptom which also has important consequences on QoL. Some participants also described a sense of social anxiety associated with discharge. Meeting new people came with the inevitability of having to explain they were not contagious; they felt the need to constantly justify why they were blowing their nose, and one patient spent a significant amount of time going to the bathroom to clean his hands because he was afraid of appearing unhygienic.

*People think it’s trivial. But it is not trivial. Everyone asks: “you still have that?”*

*We have gone to the moon; how come we cannot prevent polyps from coming back?*

(Participant 15, female, CRSwNP)

Symptomatology and disease progression varied widely between participants. That said, the severity of the impact on patients’ quality of life and the general feeling of exasperation and desperation before seeking out surgery was shared among most participants. In other words, patients seemed to elect surgery if they not only had symptoms, but if these symptoms prevented them from living a normal life.

### Objectives and expectations

As previously described, the majority of participants had a tendency to focus on a principal symptom when describing the incapacity associated with CRS. With a lot of their attention often directed towards that symptom; patients’ main objective was often to correct their most bothersome symptom.

*My objective for surgery was to eliminate the headaches. I wanted them to disappear.*

*And it worked, it really worked. But the relief only lasted 1 year and 2 months.*

(Participant 21, male, CRSsNP)

*I chose to undergo surgery because I wanted to be able to breathe and smell like before.*

*Now I can smell, and it has given me my life back.*

(Participant 22, male, CRSwNP)

Other participants described more general objectives. They wanted to improve their condition, get rid of the chronic malaise, improve their QoL, or “become normal”. Patients’ objectives and expectations both appeared inversely proportional to the amount of previous surgeries and the severity of CRS impact. In other words, patients who had only been operated once had a tendency to describe that they hoped for (or expected) a full return to normality after ESS. Patients who had undergone many surgeries had more pessimistic expectations and tended to have less ambitious objectives. Furthermore, some patients with incapacitating constant headaches or a complete incapacity to breathe from the nose stated that any small improvement would be worth going through the ESS. Only a minority of patients (5/22) hoped (or expected) all their symptoms would disappear. Other patients hoped (or expected) and improvement in one symptom in particular, or simply hoped for an improvement in QoL.

*The first surgery, I did not really have any objectives. The doctor said the polyps were so big, they came out from the nose. The second time I wanted to breath better, I wanted to have less infections and I wanted to have a lasting relief.*

(Participant 5, male, CRSwNP)

*My goals were to need less antibiotics and cortisone. Also, I wanted to get rid of my asthma. When I have nasal polyps, my asthma gets out of control.*

(Participant 15, male, CRSwNP)

### Criteria for success

Among participants, there was a clear association between expectations and satisfaction. Dissatisfied patients tended to have ambitious expectations such as a complete and prolonged cure after ESS.

*The goal I think is to feel better*. *I wanted to be 80% better … or at least 60%. I think the goal is also to feel better for as long as possible. Ideally to feel better forever, but by now I don’t really believe anymore that it’s possible. So, I would say at least 4-5 years.*

(Participant 4, female, CRSwNP)

Only one patient reported no improvement whatsoever after ESS, but several participants were not entirely satisfied. This dissatisfaction was often due to a recurrence in symptoms after a while. Patients were asked to determine what decrease in symptom magnitude would make the surgery worth it in terms of risks vs benefits. Their answers ranged from “any noticeable improvement in the main symptom” to “a complete resolution of all symptoms”. The same question was asked for time with symptomatic control. Duration ranged from “1 month” to “forever”

***Prompt: How long should you be free of symptoms for you to consider the surgery successful?***

*Had you told me 1 month, I would have still gone forth. I could not live like that anymore.*

***Prompt: How much improvement would be necessary for you to consider the surgery successful?***

*Even if it’s not perfect. Even a tiny improvement is worth it. If not for surgery, the only other solution is cortisone. And that is bad for the rest of my body.*

(Participant 22, male, CRSwNP)

## Discussion

Many interviewed participants reported significant levels of distress before ESS. Amongst our participants, the decision to undergo ESS was motivated by the desire to improve symptoms and QoL, which is consistent with findings from Soler et al. [20]. Indeed, in a group of 242 patients among which 180 patients elected to have ESS rather than medical management, there was no difference in demographic characteristics, social support, personality types or physician-patient relationship between groups. The only difference between the surgery group and the medical management group was a greater negative impact on disease-specific QoL in the ESS group as assessed by SNOT-22 score (a validated measure of CRS symptoms and health-related QoL) [21]. In light of this data, it is not surprising that our group of post-ESS participants reported a significant preoperative impact of CRS on their daily life.

Symptoms reported in our population pre-operatively were similar to those reported in the literature. Mattos et al. found that preoperatively, symptoms most often reported by CRS patients were nasal obstruction, smell/taste, discharge and sleep symptoms [22]. One notable difference in our study is that we questioned participants a posteriori rather than a priori. Despite this variation, our overall impression was similar: these symptoms seem to be the ones which lead patients to seek ESS, especially in the CRSwNP group. Some patients described symptoms they believed were associated with CRS, but which are not usually considered a part of the disease spectrum. In these instances, they may describe unrealistic goals. For example, pruritus may be due to an atopic predisposition, which is associated with CRS; that said, one does not necessarily expect this patient to resolve with the control of CRS. It is imperative that patients learn through counselling which symptoms are amenable to improvement and which are not. If patients undergo ESS with the goal of improving a symptom unrelated symptoms to CRS, disappointment is inevitable. Even rhinologic symptoms associated with CRS do not always respond to ESS. It is unclear which symptoms are most improved by ESS, but certain clinical presentations (symptom clusters) may predict overall SNOT-22 score improvements [23]. Patient-reported outcome measures (PROMs), such as the SNOT-22, are validated clinical tools that produce a numerical score which can be interpreted as an improvement or a deterioration in disease state.

One of the most interesting findings of this study is the heterogeneity of patient definitions of operative success. Patient satisfaction seems to depend greatly on the extent to which expectations and goals are met, rather than the actual process or quality of outcome. We believe that patient satisfaction remains an overly simplistic measure of success after ESS, even in the busy clinical setting. Some participants in our study reported that they would be content with any noticeable improvement, while others were expecting a complete and indefinite resolution of their symptoms. Interestingly, patients had a tendency to have the highest expectations before their first surgery. First-time ESS patients with excessively high expectations should be proactively counselled preoperatively, in the hopes of avoiding or mitigating post-operative disappointment. Conversely, patients with recurrent disease did not expect to be cured with a subsequent ESS and were more realistic regarding long-term outcomes. Counselling in these patients should focus on the increased risks of operative complications in revision ESS [24].

The great disparity between patients’ objectives, expectations and definitions of success emphasizes the importance of a thorough presurgical counselling and a strong physician-patient partnership. Last year, the *Quality Improvement Committee of the American Rhinologic Society* published a framework termed the CRS Appropriate Presurgical Algorithm (CAPA), which relies on 4 main quality metrics [25]. One of those is the occurrence of “a patient-centered discussion regarding treatment options for refractory CRS while focusing on risks and benefits, the need for long-term medical compliance and understanding of patient preferences and expectations”. Vennik et al. have recently published their extensive work on both patient and physicians’ (Otolaryngologists and primary care physicians) experiences and views on current management of CRS in the UK [26, 27]. Previously, Erskine et al. had also explored CRS patients’ experiences in both primary and secondary care, again in the UK [28]. A thorough understanding of the patient experience is essential to optimal care, especially in chronic illnesses such as CRS. This qualitative approach to the topic of ESS permitted an in-depth exploration of certain theme. This permitted us to collect rich and granular information directly from primary stakeholders.

Clinicians strive to offer the best surgical treatments, and to follow evidence-based recommendations. That said, emphasis must also be placed on tailoring the right treatment to the right patient. As we have shown, patients’ goals and expectations may vary widely. Physicians should play a role in assessing – and modulating if necessary – expectations because unrealistic goals are often synonymous with postoperative disappointment. Moreover, open and frank discussion should be geared to the severity of patients’ disease and previous experience with ESS. Preoperative counselling should thus be undertaken diligently not only for complex cases with a complicated history with CRS, but also for patients naïve to surgery or patients with seemingly minor disease. It is crucial that these patients understand that ESS is by no means an “easy fix”, that long-term positive outcomes are often contingent on continuous topical therapy, and that the process of undergoing ESS can be associated with significant discomfort and distress.

A growing body of evidence suggests an important heterogeneity both in clinical phenotypes and genetic endotypes of patients with CRS [29–31]. A greater understanding of the pathophysiology and the molecular pathways involved has stimulated efforts in targeted treatment and personalized medicine for CRS patients [32, 33]. A recent article by Grayson et al. differentiates three different CRS phenotypes: IgE-mediated central compartment atopic disease, eosinophilic CRS and non-eosinophilic CRS. This article illustrates the distinctions in clinical presentation, endoscopic, radiologic and histopathologic findings, and in treatment. In all three subtypes, the authors recommend to consider ESS, with the distinction a Draf 3 is suggested in eosinophilic CRS [34]. Despite the arrival of biologics for the treatment of CRSwNP, ESS remains the mainstay of the treatment of CRS refractory to maximal medical therapy for most patients [35, 36]. Indeed, this surgery has been shown to be efficient and more cost-effective than continuous medical therapy alone [37–40]. That said, evidence suggests certain phenotypes of CRS tend to have more favorable surgical outcomes compared to others [41]. While decision to undergo ESS is always taken in partnership with the patient, studies suggest that certain symptoms or patient characteristics are predictive of positive or negative outcomes [42]. Patient reported outcome measures, such as the Sinonasal Outcome Test (SNOT-22), or a thorough preoperative assessment can guide clinicians during patient counselling [43–46].

The limitations of this study include the subjectivity inherent to the qualitative design. Moreover, all participants were recruited from the practice of a single surgeon working in an academic tertiary referral practice. Hence, this population may not be representative of the general CRS population. That said, it provided us with insights from patients who had undergone many ESS and had a rich experience with the disease. Since the study was conducted in Montreal (Canada), interviews were conducted in patients’ preferred language. This may have led to some aspects of the experiences described by patients being lost in translation during thematic analysis. Additionally, the median time from last surgery was 12 months among participants. It is likely that patients may have forgotten some elements of their experience with ESS, though we hope the most important ones were recalled. Although all participants were followed at the time of the interview by a single surgeon, many had previously been operated at other institutions and only then referred for refractory disease. Six patients had undergone one ESS only; thus, our sample was heterogeneous enough to capture many different levels of CRS severity, or at least different timeframes. Another important limitation of this study concerns the fact that 90% of participants were Caucasian. A recent paper shows that non-white patients tend to experience more severe CRS symptoms at baseline compared to white patients. They also seem to have more significant improvements in QoL with ESS [47]. Undoubtedly, non-white patients must have different experiences with regards to ESS. We unfortunately were not able to collect and analyze these unique perspectives. Despite these limitations, we believe this study provides important insights to otolaryngologists on the patient journey before and after ESS, patients’ objectives and expectations before surgery, and patients’ perspective on the definition of an optimal outcome after surgery.

## Data Availability

Data used in the current study is available from the corresponding author on reasonable request.

## Appendix

**Question 1:** Please describe the most important aspects of your experience living with CRS.

Prompt: How would you rate them in order of importance?

Prompt: What are the best questions to assess each of these aspects?

**Question 2:** Please describe your goals before undergoing ESS.

**Question 3:** Please describe your expectations before undergoing ESS.

**Question 4:** Please list things you wish you knew going into surgery, if any.

**Question 5:** Please describe what your idea of a perfect outcome after ESS is.

**Question 6:** Please describe what would characterize a positive or a negative experience with ESS.

**Question 7:** How would you describe your experience with ESS?

Prompt: How would you rate it from 0 to 10?

Prompt: What are the strengths and weakness?

Prompt: How would you describe the final result?

**Question 8:** Please list the 5 symptoms that bother you the most in order of importance?

Prompt: How would you rate each from 0 to 10?

Prompt: How did each vary after ESS?

## Abbreviations

CRS: Chronic rhinosinusitis
CRSwNP: Chronic rhinosinusitis with nasal polyposis
CRSsNP: Chronic rhinosinusitis without nasal polyposis
ET: Eustachian tube
ESS: Functional endoscopic sinus surgery
QoL: Quality of life
